# Feasibility of a laboratory-based protocol for measuring energy expenditure and accelerometer calibration in adults with intellectual disabilities

**DOI:** 10.1186/s40814-024-01512-5

**Published:** 2024-06-22

**Authors:** A. M. McGarty, V. Penpraze, P. M. Dall, C. Haig, L. Harris, C. A. Melville

**Affiliations:** 1 School of Health & Wellbeing, University of Glasgow, Clarice Pears Building, 90 Byres Road, Glasgow, G12 8TB Scotland; 2 School of Life Sciences, University of Glasgow, Sir James Black Building, Glasgow, G12 8QQ Scotland; 3 School of Health & Life Sciences, Glasgow Caledonian University, Cowcaddens Road, Glasgow, G4 0BA Scotland

**Keywords:** Intellectual disabilities, Adults, Sedentary behaviour, Physical activity, Energy expenditure, Laboratory-based research, Accelerometer calibration

## Abstract

Adults with intellectual disabilities experience numerous health inequalities. Targeting unhealthy lifestyle behaviours, such as high levels of sedentary behaviour and overweight/obesity, is a priority area for improving the health and adults with intellectual disabilities and reducing inequalities. Energy expenditure is a fundamental component of numerous health behaviours and an essential component of various free-living behaviour measurements, e.g. accelerometry. However, little is known about energy expenditure in adults with intellectual disabilities and no population-specific accelerometer data interpretation methods have been calibrated. The limited research in this area suggests that adults with intellectual disabilities have a higher energy expenditure, which requires further exploration, and could have significant impacts of device calibration. However, due to the complex methods required for measuring energy expenditure, it is essential to first evaluate feasibility and develop an effective protocol. This study aims to test the feasibility of a laboratory-based protocol to enable the measurement of energy expenditure and accelerometer calibration in adults with intellectual disabilities.

We aimed to recruit ten adults (≥ 18 years) with intellectual disabilities. The protocol involved a total of nine sedentary, stationary, and physical activities, e.g. sitting, lying down, standing, and treadmill walking. Each activity was for 5 min, with one 10 min lying down activity to measure resting energy expenditure. Breath by breath respiratory gas exchange and accelerometry (ActiGraph and ActivPAL) were measured during each activity. Feasibility was assessed descriptively using recruitment and outcome measurement completion rates, and participant/stakeholder feedback.

Ten adults (*N* = 7 female) with intellectual disabilities participated in this study. The recruitment rate was 50% and 90% completed the protocol and all outcome measures. Therefore, the recruitment strategy and protocol are feasible.

This study addresses a significant gap in our knowledge relating to exercise laboratory-based research for adults with intellectual disabilities The findings from this study provide essential data that can be used to inform the development of future protocols to measure energy expenditure and for accelerometer calibration in adults with intellectual disabilities.

## Key feasibility messages

- ***What uncertainties existed regarding the feasibility?***
  - Can we recruit adults with intellectual disabilities to a laboratory-based study?
  - Can adults with intellectual disabilities complete the protocol and measurements required for measuring energy expenditure and accelerometer calibration?
- ***What are the key feasibility findings?***
  - It is feasible to recruit adults with intellectual disabilities to a laboratory-based study.
  - It is feasible for adults with intellectual disabilities complete the protocol and measurements required for measuring energy expenditure and accelerometer calibration.
- ***What are the implications of the feasibility findings for the design of the main study?***
  - No changes are required to the protocol when designing the main study. Ensuring flexibility within the protocol will aid compliance, e.g., self-selected treadmill speeds and protocol duration.
  - The main study should aim to recruit a more even gender split to investigate gender-specific differences in energy expenditure and accelerometer calibration.

## Introduction

Adults with intellectual disabilities experience numerous health inequalities, such as increased risk of non-communicable diseases and obesity [1–3]. As a result, adults with intellectual disabilities have a life expectancy that is 20 years less than people without intellectual disabilities [4]. One contributing factor to these health inequalities and lower life expectancy is unhealthy lifestyle behaviours, which has been highlighted as a key area for future research focus [1]. Various behaviours come under the umbrella term of “lifestyle behaviours”, including sedentary behaviour and physical activity. These are important behaviours to understand as being physically active and having low sedentary time have numerous positive health outcomes, such as reduced risk of cardiovascular disease and obesity [5].

Physical activity and sedentary behaviour can be measured with various tools and outcomes [6]. For example, subjective measures can include self-report questionnaires to report on frequency and type of physical activities and sedentary behaviours conducted. Device-based measures include pedometers to measure steps and accelerometers to measure time spent sedentary and in different activity intensities. However, the recall and cognitive requirements for subjective measures raises validity questions for use in adults with intellectual disabilities, and therefore device-based measures are more appropriate for the needs of this group [7]. Accelerometers are the most commonly used device to measure total physical activity and sedentary behaviour in adults with intellectual disabilities, with free-living measurement protocols feasible for adults with intellectual disabilities [7, 8].

A significant limitation with accelerometers is in relation to data interpretation, as these devices measure acceleration that is then converted into a relevant outcome, such as intensity, by applying algorithms that are based on energy expenditure. The interpretation of accelerometer data highlights a significant two-fold limitation in this field of research; a lack of knowledge of actual energy expenditure and lack of calibration studies. Firstly, little is known about the energy expenditure of adults with intellectual disabilities during sedentary behaviour and physical activity. Two laboratory-based studies have investigated energy expenditure in adults with intellectual disabilities, with both reporting that adults with intellectual disabilities had a higher energy expenditure during various activities, compared to adults without intellectual disabilities [9, 10]. Furthermore, a follow-on to the Iwoaka et al. study [9], as well as the study by Lante et al. [10], suggested that the higher energy expenditure was due to excessive body movements, e.g. fidgeting, rather than limitations with skeletal muscles [11].

These initial findings of higher energy expenditure in adults with intellectual disabilities could have significant implications for measuring sedentary behaviour and physical activity using accelerometers, which requires further investigation. However, potential issues were raised in these studies about the feasibility of the protocol and measurements used in laboratory-based research. For example, in the study by Lante et al. [10] participants required up to six familiarization sessions prior to data collection, with 36% of the original sample withdrawn due to issues with completing the protocol, such as discomfort with the equipment, safety, and participant anxiety. Therefore, this highlights the need to be aware of feasibility when conducting laboratory-based physical activity and sedentary behaviour research in adults with intellectual disabilities.

The second gap in knowledge within this field of research is that no accelerometer calibration studies have been conducted in adults with intellectual disabilities. To increase validity, data interpretation methods, such as cut points, should be population-specific to account for biomechanical or physiological differences between populations. Accelerometer cut points have been calibrated for various populations, such as adults with multiple sclerosis or Parkinson’s, as well as children with intellectual disabilities [12–14]. However, due the lack of calibration in adults with intellectual disabilities, researchers, in general, have to use data interpretation techniques calibrated in people without intellectual disabilities [7]. However, given the differences in energy expenditure described previously, this would lead to measurement error and reduce the validity of results.

Therefore, to address these gaps in the literature, there is a need to measure energy expenditure during sedentary behaviour and physical activity to compare to these previous studies and to enable device calibration. These two aims can be achieved using a laboratory-based protocol with a criterion measure of energy expenditure, such as indirect calorimetry. However, as no protocols exist for device calibration for adults with intellectual disabilities, and to ensure the safety and appropriateness of the protocol for measuring energy expenditure, it is essential to conduct a feasibility study prior to conducting a full-scale study. Therefore, the following research questions will be investigated:

1. Is it feasible to recruit adults with intellectual disabilities to a laboratory-based study to measure energy expenditure and for accelerometer calibration?
2. Is it feasible for adults with intellectual disabilities to complete the protocol?
3. Is it feasible for adults with intellectual disabilities to complete all outcome measurements?

## Methods

### Ethical considerations

This study was approved by the Medical, Veterinary, and Life Sciences College ethics committee, University of Glasgow and aligns with the ethical principles of the Declaration of Helsinki. All information and consent forms were in an easy read format and written informed consent was obtained from all participants.

### Participants and recruitment

The eligibility criteria for this study were: adults (aged ≥ 18 years) who have intellectual disabilities, defined as significant limitations in both intellectual functioning and adaptive behaviour that originates during the developmental period [15]. The exclusion criterion were people who could not ambulate independently. Participants were recruited through community-based organisations for people with intellectual disabilities in the Greater Glasgow area. The aim of this study was to recruit a convenience sample of *N* = 10 participants through a structured recruitment strategy. A £20 gift voucher was given to all participants as a thank-you for their time. The recruitment strategy involved making initial contact with three community-based organisations that support people with intellectual disabilities via phone or email, then arranging to attend a group meeting or event to present on the study and enable discussion about participation. Recruitment began in February 2020, was subsequently suspended in March 2020 due to the COVID-19 pandemic, then recommenced in March 2022 until October 2022.

### Protocol

This feasibility study was conducted in three stages: familiarisation (e.g. showing participants the laboratory), preparation (e.g. practicing procedures), and data collection [16]. It was envisaged that these stages would be conducted during a single session to reduce the burden on participants. However, as previous laboratory-based studies in adults with intellectual disabilities have required up to six familiarisation/ preparation sessions prior to data collection [10], additional sessions were offered to ensure participants are comfortable with the procedures prior to data collection.

Data collection involved four sedentary activities, one stationary activity, and three physical activities. The sedentary activities involved participants sitting down, reclining, and lying down; these activities are representative and common sedentary behaviours [17]. In addition, participants completed a lying at rest activity to enable resting energy expenditure to be measured. The stationary activity involved standing unaided (where appropriate), and the physical activities involved treadmill walking at self-selected speeds, which represented slow, normal, and fast walking. Participants completed each activity for 5 min (with the exception of lying at rest, which was conducted for 10 min), with short breaks in-between activities.

### Measures

#### Demographic

Participants (with support from a carer, where necessary) were asked to report their date of birth, gender, level of intellectual disabilities, cause of intellectual disabilities, and any cooccurring conditions.

#### Anthropometric

Anthropometric measurements were measured in accordance with the International Standards for Anthropometric Assessment [18]. Height was measured to the nearest 0.1 cm using a stadiometer (Seca Scales, Hamburg, Germany), and weight was measured to the nearest 0.1 kg using digital scales (Seca Scales, Hamburg, Germany). Body mass index (BMI) was calculated based on these measurements [weight/height^2^ (kg/m^2^)]. Measurements were conducted twice to produce a mean value whilst participants were wearing light clothing and no shoes.

#### Sedentary behaviour and physical activity

The ActiGraph wGT3X + (ActiGraph LLC, Pensacola, FL, USA) is a small triaxial accelerometer which measures acceleration of the body across the vertical, horizontal, and perpendicular axes during movement. Prior to the session, the accelerometers were initialized in accordance with manufacturer specifications and set to record at a frequency of 100Hz. The device was worn around the waist, positioned at the hip (at the iliac crest) and attached using an elastic belt. Participants were also asked to wear an ActivPAL3™ monitor (PAL Technologies, Glasgow, UK), which is a valid method to measure sedentary behaviour in people without intellectual disabilities [19]. This small monitor uses an accelerometer to measure limb inclination and physical activity, and was secured onto the right anterior mid-line of the right thigh with a hypoallergenic patch (PAL stickie), and covered with a waterproof dressing (Opsite Flexifix). Participants were asked to wear the ActivPAL and ActiGraph throughout the protocol.

#### Oxygen consumption

The Ultima CPX (Medical Graphics, MN, USA), which analyses expired gases on a breath-by-breath basis, was used to measure respiratory gas exchange. Airflow, ventilatory volume, and gas analysers were calibrated using standard measures in accordance with manufacture guidelines prior to each measurement. Participants wore a prevent® (Medical Graphics, MN, USA) material mask which covered their nose and mouth. This was attached directly to a bidirectional flow meter, a sampling line, and measurement sensor. Oxygen uptake was measured for each protocol activity, with the mask removed in between activities.

#### Direct observation

To enable potential “fidgeting” movements to be observed, all sessions were video recorded using a GoPro HERO 3 + camera. A separate section was included in the consent form to enable participants to consent or not consent to video recording during the session.

### Data analysis

Data were analysed using SPSS 27 IBM statistical package (SPSS IBM, New York, NY, USA). Descriptive statistics were calculated for sex, age, height, weight, and body mass index (BMI), with means, standard deviations (SD). Recruitment rates were calculated based on the number of people approached compared to the number of people recruited, calculated as a percentage. Feedback from staff and eligible people relating to the recruitment strategy will be presented descriptively. Protocol and outcomes measures completion rates and reasons for non-completion were recorded via observation and participant feedback and presented as descriptive statistics.

## Results

### Participant characteristics

Ten adults (7 females and 3 males) participated in this study. The age of participants ranged from 19 – 66 years, with a mean age of 41.00 (SD = 17.24) years. Levels of intellectual disabilities ranged from mild to moderate, with one participant having autism in addition to intellectual disabilities. Mean BMI was 31.11 kg/m^2^ (SD = 5.00; range: 24.55 – 40.83 kg/m^2^).

### Research question 1: recruitment

The COVID-19 pandemic impacted recruitment as two of the three organisations contacted when the study initially commenced in 2020 had discontinued by the time the study restarted January 2022. The third organisation was still willing to support recruitment, although they discussed that their priority was to support their members to overcome the impacts of the pandemic, rather than research. This organisation also noted that their members were concerned about face-to-face interactions. Therefore, recruitment was suspended for a further 3 months until March 2022 to enable the group and their members to become more comfortable with being involved in research. Once recruitment started, *N* = 20 information packs were distributed to group members. Of these, *N* = 10 agreed to participate, giving a recruitment rate of 50%.

Feedback from the organisation and members was that face-to-face interactions were still a concern for some people who were at a higher risk from COVID-19 infection. To mitigate this, the option was given to discuss the project using an online video call; for in-person meetings, all relevant safety procedures were adhered to, e.g. mask wearing. In addition, this organisation described that some participants were concerned about travelling to the University for data collection. Therefore, full support was given in relation to transport, e.g. assisting with planning public transport or arranging taxis. Two participants attended together, four attended with a support worker, and four attended on their own. Feedback from participants was that that the £20 voucher offered was an incentive to take part.

### Research question 2: protocol

The full results relating to completion of the protocol are presented in Table 1. Nine participants (90%) completed the protocol as intended. One participant withdrew during data collection due to a personal reason not related to the study. All participants completed the protocol during one session. Time to complete the protocol ranged from 45 – 79 min (M = 61, SD = 9 min). Mean self-selected treadmill speeds for the slow, normal, and fast walking speeds were: 1.39 km/h (SD = 0.26; range = 1.10 – 1.90 km/h), 2.36 km/h (SD = 0.38; range = 1.70 – 2.90 km/h), 3.32 km/h (SD = 0.49; range = 2.40 – 4.0 km/h), respectively. Therefore, as the completion rate was high and no issues were identified, this protocol is feasible for adults with intellectual disabilities to complete.

**Table 1:** Summary of feasibility results for each participant

**ID**	**BxB**	**activPAL**	**ActiGraph**	**Activities**	**Direct observation**
1	Completed	Completed	Completed	Completed	Completed
2	Completed	Completed	Completed	Completed	Completed
3	Not completed^a^	Not completed^a^	Not completed^a^	Not completed^a^	Not completed^a^
4	Completed	Completed	Completed	Completed	Completed
5	Not completed^b^	Completed	Completed	Completed	Completed
6	Completed	Completed	Completed	Completed	Completed
7	Completed	Completed	Completed	Completed	Completed
8	Completed	Completed	Completed	Completed	Completed
9	Completed	Completed	Completed	Completed	Completed
10	Completed	Completed	Completed	Completed	Completed

Completed: fully completed

Not completed^a^: Participant withdrew during data collection for a personal reason not related to the study

Not completed^b^: Not completed due to study reason; participant found it difficult to breath whist wearing the mask

### Research question 3: outcome measures

The full completion rates for the outcome measures are presented in Table 1. Ten (100%) participants consented to measurements and *N* = 9 complied with the measurement protocols for the ActiGraph, activPAL, and direct observation. One participant chose not to continue with the oxygen consumption measurement after the first activity. Feedback from this participant was that they had severe asthma and found it difficult to comfortably breathe whilst wearing the mask. This participant continued with the remainder of the protocol, except for this measurement. The participant who withdrew from participation complied with all outcome measures prior to their withdrawal.

## Discussion

This study is the first to investigate the feasibility of a laboratory-based protocol for measuring energy expenditure and accelerometer calibration in adults with intellectual disabilities. This study demonstrated that the protocol and measurements under investigation were feasible and that no changes are required to progress to a full-scale study. There are, however, some important findings from this study that require further discussion.

In comparison to previous research, the compliance rates in this study were higher. No participants were withdrawn or dropped out due to the protocol; one participant withdrew during data collection for a personal reason unrelated to the study and one person could not continue to an asthma flare-up. Furthermore, all participants completed the study phases (familiarization, preparation, and data collection) in one session. In comparison with previous research, 36% of recruited participants withdrew due to feasibility issues and an average of four (range: 2–6) familiarization sessions were required prior to data collection [10]. However, the measurements conducted within Lante et al. [10] were more invasive, such as basal metabolic rate measurements that required a minimum 10 h fast prior to data collection, which were not conducted in the present study. Therefore, this could highlight a trade-off that may be required in this field of research, where priorities have to be considered and only the most necessary procedures and outcome measures included. For example, as energy expenditure during physical activities and sedentary behaviour were the primary outcomes required for the present study, more invasive and in-depth measurements, such as basal metabolic rate, were not required. Therefore, this could have positively contributed to the compliance rates.

With the aim of increasing compliance and for participant safety and comfort, the present study used self-selected walking speeds for the physical activities. These speeds, which were intended to represent slow, normal, and fast walking, were lower than the walking speeds identified in the compendium of physical activities for people without intellectual disabilities [20]. However, this is consistent with Lante et al. [10], as participants in this study experienced higher intensity activity at slower speeds than people without intellectual disabilities, i.e. slow walking for people without intellectual disabilities was considered to be moderate intensity walking for adults with intellectual disabilities. Similarly, Iwaoke et al. [9] observed that adults with intellectual disabilities had a higher stride rate than the control group when walking at the same speeds. These finding has also been confirmed in the wider field of research, with Agiovlasitis et al. [21] demonstrating that adults with intellectual disabilities, specifically Down syndrome, required a higher level of oxygen uptake for walking compared to adults without intellectual disabilities. Therefore, this confirms the importance of further examining energy expenditure of physical activity in adults with intellectual disabilities. Furthermore, in highlighting the greater variability in the energy demands of walking, this also suggests that walking speeds should be self-selected to ensure that the desired intensities are achieved for each individual.

The present study also included an uneven gender split of participants, with seven females and three males. Although this should not impact the feasibility results, previous research has reported differences in energy expenditure between men and women with intellectual disabilities, with higher rates reported for males [10]. This is consistent with research involving people without intellectual disabilities, where greater variability in energy expenditure has been reported for men compared to women [22]. Therefore, a more equal gender split should be sought in future research relating to energy expenditure to enable investigation of gender differences in adults with intellectual disabilities. In addition, this study identified a potential impact relating to cooccurring conditions, with one participant not able to complete the protocol due to having asthma. As people with intellectual disabilities have a higher prevalence of respiratory conditions [23], this could impact completion rates for adults with intellectual disabilities who have a cooccurring respiratory condition.

This study was also significantly impacted by the COVID-19 pandemic. This primarily impacted recruitment, although the procedures put in place were effective in limiting the impact. This highlighted the need to be flexible with the needs and wants of people with intellectual disabilities in terms of ensuring their continued safety and protection from infection, such as mask wearing during data collection, as adults with intellectual disabilities are at a greater risk from infection [24]. A wider concern also identified in this study was the decline of community support opportunities available to adults with intellectual disabilities since the COVID-19 pandemic. From a research perspective, this could impact the opportunities to connect with people with intellectual disabilities and involve people in future research. But, more importantly, community participation is an important way for people with intellectual disabilities to get various support, to develop social relationships, and to be physically active [25, 26]. Therefore, reduced support for participation in community groups, or less availability of community groups, could have numerous negative impacts on various physical, social, and mental health outcomes.

The primary strength of this study was the investigation of feasibility to ensure a safe and effective protocol was developed, which reduces unnecessary participant burden and enables data collection in future research to be as effective as possible. Not without limitation, the sample for this study were recruited through community organisations. Therefore, this could introduce bias into the sample as this group are more likely to have milder levels of intellectual disabilities, thus reducing generalizability of these findings to people with more severe levels of intellectual disabilities. Although the sample size for this study was sufficient to answer the research questions under investigation, it is too small a sample to provide representative data. Therefore, no data from the outcome measures have been presented; however, these will be included with data from a future full-scale study.

In conclusion, this study has demonstrated that it is feasible to conduct a combined laboratory-based protocol for measuring energy expenditure and accelerometer calibration in adults with intellectual disabilities. This study found high completion rates across all protocol activities and outcome measures, with a recruitment rate of 50%. These findings therefore add important new knowledge to the field of conducting laboratory-based research with people with intellectual disabilities.

## Data Availability

The datasets generated and/or analysed during the current study are not publicly available due to restrictions with the ethical approval but are available from the corresponding author on reasonable request.

## Abbreviations

BMI: Body mass index
M: Mean
SD: Standard deviation
